# A single intake of flavanol-rich cocoa improves inhibitory executive process under cognitive fatigue during aerobic exercise in men: a randomized, double-blind, placebo-controlled crossover trial

**DOI:** 10.1007/s00213-025-06826-7

**Published:** 2025-06-10

**Authors:** Hayato Tsukamoto, Sota Yoneya, Takahiro Koyama, Asuka Suzuki, I Wayan Yuuki, Kento Dora, Takeshi Hashimoto

**Affiliations:** 1 Faculty of Sport Sciences, Waseda University, 2-579-15 Mikajima, Tokorozawa, Saitama, 359-1192 Japan; 2 Faculty of Sport and Health Science, Ritsumeikan University, Shiga, Japan; 3 Institute of Advanced Research for Sport and Health Science, Ritsumeikan University, Shiga, Japan

**Keywords:** Antioxidant, Arousal, Brain-derived neurotrophic factor, Decision-making, Mood, Perceived exertion, Reaction time, Supplement

## Abstract

**Rationale:**

Although cognitive fatigue commonly occurs during sports, effective strategies to improve it during exercise have not been established.

**Objectives:**

This study determined whether high-cocoa flavanol (HCF) consumption improves reaction time and inhibitory executive function impairments during prolonged cognitive load combined with aerobic exercise.

**Methods:**

In this randomized crossover study, 18 healthy males (22 ± 2 years) participated in both low-cocoa flavanol (LCF) and HCF trials. Double-blinded capsules (LCF 50 mg and HCF 500 mg) were consumed 1 h before a 50-min cognitive exercise dual-tasking protocol, which included a color-word Stroop task (CWST) and moderate-intensity cycling. The CWST assessed reaction time and reverse-Stroop interference score as indicators of inhibitory executive process.

**Results:**

Reaction time (LCF 774 ± 146 ms *vs.* HCF 731 ± 101 ms, *P* < 0.01) and reverse-Stroop interference score (LCF 6.2 [3.2–15.5] *vs.* HCF 4.6 [1.2–11.4], *P* < 0.01) were significantly better 1 h after HCF consumption than after LCF consumption, indicating that HCF improved both reaction time and the inhibitory executive process at rest. During the 50-min cognitive-exercise dual-tasking protocol, HCF consumption resulted in faster reaction time (LCF 712 ± 122 ms *vs.* HCF 685 ± 111 ms, *P* < 0.05) and better inhibitory executive process (LCF 8.4 ± 5.0 *vs.* HCF 6.6 ± 3.5, *P* < 0.05) compared to those following LCF consumption.

**Conclusions:**

These findings suggest that flavanol-rich cocoa may improve reaction time and inhibitory executive process impaired by cognitive fatigue during aerobic exercise.

## Introduction

Executive function is a critical cognitive process that supports daily activities across various populations. It encompasses three key aspects: shifting mental sets, monitoring and updating working memory representations, and inhibiting prepotent responses (Miyake et al. 2000). For example, executive function regulates both the speed and accuracy of decision-making, which are essential for behavioral performance (Funahashi 2001). However, prolonged cognitive activity can acutely impair decision-making speed and/or accuracy, leading to cognitive fatigue—a state of mental exhaustion that affects executive function (Wang et al. 2014; Tsukamoto et al. 2022). Indeed, extended engagement in the color-word Stroop task (CWST)—a well-established test of inhibitory executive control (Stroop 1935; Song and Hakoda 2015)—not only increases state mental fatigue but also slows reaction time and introduces significant intra-individual variability in response speed at rest (Wang et al. 2014; Tsukamoto et al. 2022).

Interestingly, Scholey et al. (2010) found that the negative impact of prolonged cognitive effort on state mental fatigue and cognitive function at rest could be mitigated by a single consumption of flavanol-rich cocoa, which contains antioxidant compounds derived from the seeds of the fruit of the *Theobroma cacao* tree (Miller et al. 2008). Although the underlying physiological mechanism(s) remain poorly elucidated, consuming high cocoa flavanol (HCF) appears to be a promising nutritional strategy for reducing cognitive fatigue at rest. It is known that cognitive function can be impaired by exaggerated oxidative stress (Ando et al. 2020). Given that oxidative stress is increased by psychological stress such as examinations in university students (Sivonová et al. 2004), it is assumed that the antioxidant properties of HCF may contribute to reduced cognitive fatigue. Besides, Rendeiro et al. (2013) reported that the antioxidant properties of HCF stimulate the secretion of brain-derived neurotrophic factor (BDNF) in animal brains, which is associated with certain cognitive functions.

The inhibitory executive process, along with the speed and accuracy of decision-making, may be key factors contributing to enhanced sports performance (*e.g.*, football and basketball) and are considered as important as technical skills (*e.g.*, ball control), athlete physique (*e.g.*, height and weight), and physiological components (*e.g.*, strength and cardiorespiratory fitness) (Meylan et al. 2010; Scharfen and Memmert 2019). In addition to cognitive activity (*e.g.*, prefrontal cortex activation (Wagner et al. 2001)), aerobic exercise (*e.g.*, motor cortex activation (Fontes et al. 2020)) also induces a subjective feeling fatigue as state mental fatigue (Tsukamoto et al. 2017). Nonetheless, aerobic exercise has been shown to acutely improve cognitive functions, such as decision-making quality and inhibitory executive process, despite increasing state mental fatigue (Wiśnik et al. 2011; Ogoh et al. 2014; Tsukamoto et al. 2016a, b, 2017; Pontifex et al. 2019). For instance, reaction time, measured by CWST, gradually shortens during aerobic exercise performed for 50 min (Ogoh et al. 2014). However, when compared to aerobic exercise alone, we have reported that a further increase in state mental fatigue during aerobic exercise combined with a 50-min prolonged cognitive effort (*i.e.*, motor-cognitive dual-task with an ‘additional’ cognitive task (Herold et al. 2018)) can slow the CWST-measured reaction time (*i.e.*, slow decision-making speed) and increase the interference score of the CWST (*i.e.*, inhibitory executive process impairment), even during aerobic exercise (Tsukamoto et al. 2022). These findings suggest that cognitive fatigue-related cognitive decline can occur even during sports when prolonged cognitive activity is required and may be one of the factors that impair sporting performance. To the best of our knowledge, no study has identified strategies for improving cognitive fatigue-related cognitive decline during aerobic exercise. Hence, it is worth exploring the potential positive impact of feasible strategies (*e.g.*, supplements) on decision-making quality and the inhibitory executive process during prolonged cognitive load combined with aerobic exercise.

Given that HCF mitigates prolonged cognitive load-induced state mental fatigue at rest (Scholey et al. 2010), it is plausible that state mental fatigue resulting from prolonged cognitive load during aerobic exercise would be lower after HCF than after low-cocoa flavanol (LCF) consumption (*i.e.*, placebo control). Moreover, we have previously demonstrated that HCF consumption can improve decision-making speed and inhibitory executive process after aerobic exercise in men (Tsukamoto et al. 2018). Based on these findings, we hypothesized that HCF could improve cognitive fatigue-related impairments in decision-making speed and inhibitory executive process during prolonged cognitive load in conjunction with aerobic exercise. To test this hypothesis, we conducted a randomized, double-blind, placebo-controlled crossover study in healthy male participants to compare decision-making quality and inhibitory executive process during prolonged cognitive load combined with aerobic exercise between LCF and HCF trials. In addition, to explore the physiological mechanism(s) underlying the impact of HCF on behavioral performance, we assessed circulating thiobarbituric acid reactive substances (TBARS) as a biomarker of oxidative stress and BDNF levels.

## Materials and methods

### Ethics and participants

All procedures were approved by the Ethics Committee of Ritsumeikan University (BKC-LSMH-2021–075) and adhered to the *Declaration of Helsinki*, except for database registration. A priori sample size calculation (using G*Power) indicated that assuming variability in the inhibitory executive process in response to completing a prolonged CWST during exercise compared to aerobic exercise alone (*i.e.*, the effect of prolonged cognitive activity on cognition during aerobic exercise) (Tsukamoto et al. 2022), a sample size of 17 participants would provide a statistical power of 80% at an α level of 0.05. To account for potential participant drop-out, we randomly recruited 18 healthy males (aged 22 ± 2 [range 20–25] years; height 172.5 ± 6.7 cm; body mass 65.6 ± 4.7 kg; peak oxygen uptake [VO_2_ peak] 46 ± 4 mL/kg/min) to participate in the study. All participants provided written informed consent before participation. Inclusion criteria required participants to be free from neurological, cardiovascular, or pulmonary disorders, not taking any medication, and to be nonsmokers. Participants were instructed to refrain from alcohol and caffeine consumption, as well as strenuous physical activity, for 24 h before each experimental visit. Each participant was requested to abstain from consuming food for 12 h prior to each experiment. Experiments were conducted in a controlled environment at 22–24 °C.

### Experimental conditions and procedure

Participants initially completed a 10-min preliminary session to familiarize themselves with the cognitive assessment (*i.e.*, the CWST score reaches asymptote) during cycling exercise (*i.e.* a dual task of prolonged cognitive activity and aerobic exercise), and their cardiorespiratory fitness was assessed (Visit 1). Subsequently, two separate trials (*i.e.*, LCF and HCF) were completed on separate days (Visits 2 and 3) in a randomized, counterbalanced manner (a within-participants crossover design (Pontifex et al. 2019)). Experimental sessions took place between 08:00 and 10:00 am, with each visit separated by a minimum of 72 h.

**Fig. 1:**
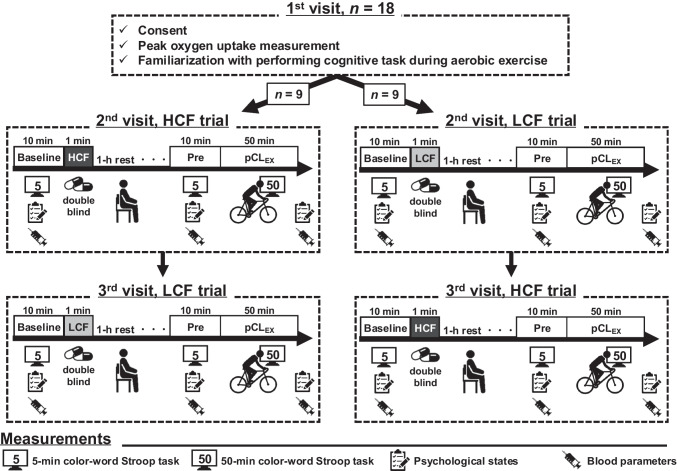
Schematic overview of the experimental design. HCF, high cocoa flavanol; LCF, low cocoa flavanol

On experimental days (Visits 2 and 3), an 18*G* cannula was inserted into the cephalic vein of the non-dominant arm for blood sampling upon arrival. After a 10-min rest, participants performed a 5-min CWST to assess baseline decision-making speed, accuracy and inhibitory executive process. Next, the heart rate (HR), psychological state, and blood parameters were measured. After confirming minimal day-to-day variation in the baseline CWST score, participants were given double-blinded capsules (LCF 50 mg or HCF 500 mg) (Scholey et al. 2010; Tsukamoto et al. 2018). It is known that plasma flavanol levels (*e.g.*, epicatechin) peak 2 h after HCF consumption, with increases observable 30 min after intake (Holt et al. 2002; Schroeter et al. 2006). Cognitive improvements are reported to occur 30 min to over 2 h after HCF consumption (Scholey et al. 2010; Tsukamoto et al. 2018). Accordingly, 1 h post-consumption (Tsukamoto et al. 2018), HR, psychological state, 5-min CWST scores, and blood parameters were measured before participants underwent a 50-min cognitive load in conjunction with aerobic exercise protocol. Subsequently, participants continuously performed the CWST while engaging in moderate-intensity cycling exercises for 50 min (Tsukamoto et al. 2022). After the prolonged cognitive activity during aerobic exercise, HR, psychological state, and blood parameters were re-assessed (Fig. 1).

### Measurements

#### Cardiorespiratory fitness

VO_2_ peak was determined using an exercise bike (95 C Inspire Upright Lifecycle; Life Fitness, Tokyo, Japan). The exercise test began at a workload of 60 W for 1 min, followed by an incremental increase of 15 W/min until the participant could no longer maintain a pedaling cadence of 60 rpm (Tsukamoto et al. 2019, 2022). Throughout the test, the expired gas fractions were measured continuously using an online breath-by-breath gas analysis (AE-310S; Minato Medical Science, Osaka, Japan). VO_2_ peak was defined as the highest 30-s average recorded before exhaustion and was used to calculate moderate-intensity exercise levels for the relevant experimental conditions (60%VO_2_ peak 149 ± 16 W).

#### HR

HR was monitored at rest and during prolonged cognitive activity combined with aerobic exercise using a telemetry system (RS400 and H10; Polar Electro, Kempele, Finland).

#### Psychological states

The Visual Analog Scale (VAS) included questions on five psychological types that assessed state mental fatigue, the ability to concentrate on the CWST, motivation for the CWST, mental stress, and irritation. Each VAS score ranged from 0 (not at all) to 100 (extremely), with participants indicating their responses by marking a line on the scale (Tsukamoto et al. 2016a, b, 2017, 2022). Arousal levels were evaluated using the Felt Arousal Scale (FAS), which ranges from 1 (low arousal) to 6 (high arousal) (Svebak and Murgatroyd 1985). Additionally, participants provided a rating of perceived exertion (RPE) using the Borg 6–20 scale, which ranges from 6 (no exertion) to 20 (maximal exertion), to estimate the effort expended during prolonged cognitive activity and aerobic exercise (Borg 1982).

#### Quality of decision-making and inhibitory executive process

The speed and accuracy of decision-making and inhibitory executive process were evaluated using the CWST (Stroop 1935), a widely used paradigm for assessing cognitive performance. CWSTs lasting 5 and 50 min were programmed in SuperLab (Cedrus, San Pedro, CA, United States) (Tsukamoto et al. 2022). The word stimuli consisted of four color names—red, blue, yellow, and green—presented in Japanese characters. Participants completed three types of CWST: 1) a congruent task, an easily facilitated task where color names were displayed in the matching-colored text; 2) a neutral task, a control condition where color names were displayed in the black text; and 3) an incongruent task, an interference task where color names were displayed in the mismatched-colored text. Each stimulus was presented for 1500 ms following a 500-ms fixation cross in a counterbalanced random order with interstimulus intervals of 1, 3, or 5 s to prevent participants from predicting the timing of subsequent tasks (Tsukamoto et al. 2022). Specifically, the 5-min CWST consisted of 60-word stimuli (20 stimuli per task type), while the 50-min CWST included 600-word stimuli (200 stimuli per task type). A color-labeled keyboard (RB-540, Cedrus, San Pedro, CA, United States) was used, and participants were instructed to press the key corresponding to the color text meaning of the stimulus word. The average reaction time, standard deviation (SD) of reaction time (*i.e.*, intra-individual reaction time variability), and total number of errors were assessed using data from both the 5-min CWST (*i.e.*, baseline and before prolonged cognitive load with aerobic exercise protocol) and 50-min CWST (*i.e.*, during prolonged cognitive load with aerobic exercise protocol). To evaluate inhibitory executive processes, reverse-Stroop interference scores were calculated as the average reaction time [(incongruent task—neutral task)/neutral task)] × 100 (Tsukamoto et al. 2022).

#### Blood parameters

Blood samples were collected into vacutainers containing EDTA-2 Na (Terumo Inc., Tokyo, Japan) for plasma samples and in 1-mL syringes for measuring glucose (photometry; Terumo Inc., Tokyo, Japan), lactate (Lactate Pro2; Arkray, Kyoto, Japan), hemoglobin (Hb201; HemoCue, Ängelholm, Sweden), and hematocrit. In addition to glucose, circulating lactate is known as an important energy substrate for the brain, particularly during aerobic exercise (Hashimoto et al. 2021). Hemoglobin and hematocrit levels were used to calculate changes in relative plasma volume (Dill and Costill 1974; Tsukamoto et al. 2019), which can indicate dehydration, a factor known to affect cognition (Watanabe et al. 2024). Plasma samples were also used to measure TBARS and BDNF levels. Each vacutainer was gently mixed and centrifuged at 3,000 × *g* for 15 min at 4°C immediately after collection. TBARS (Cayman Chemical, Ann Arbor, MI, USA) and BDNF (R&D Systems, Minneapolis, MN, USA) levels were quantified using ELISA kits. To ensure complete platelet removal before BDNF analysis, we thawed and centrifuged plasma samples again at 10,000 × *g* for 10 min at 4 °C (Tsukamoto et al. 2021).

### Statistical analysis

Data are expressed as means ± SD when normality was confirmed using Shapiro–Wilk tests (IBM SPSS Statistics version 30, Armonk, NY, United States). Non-normally distributed data are expressed as medians (interquartile ranges [IQR]). All individual and raincloud plots were generated using JASP software (version 0.16.4, University of Amsterdam, Netherlands).

Baseline data between the two trials were initially compared using a paired two-tailed *t*-test for normally distributed data and the Wilcoxon signed-rank test for non-normally distributed data. Next, a generalized linear mixed model (GLMM) with random effects for baseline levels was used to analyze the reproducibility of the impacts of HCF compared to LCF. The RPE in response to aerobic exercise during prolonged cognitive activity was analyzed using the Wilcoxon signed-rank test. Given the differing durations of the CWST before and during prolonged cognitive activity in conjunction with aerobic exercise (*i.e.*, 20 stimuli and 200 stimuli for each CWST), along with single- and dual-tasking, a GLMM with random effects for baseline scores was used to compare the 50-min data during/after prolonged cognitive activity in conjunction with aerobic exercise between the LCF and HCF trials. The statistical significance level was set at *P* < 0.05. The effect size was estimated using Cohen’s *d* for normally distributed data and *r* for non-normally distributed data. Cohen’s *d* strength of effect size was interpreted as weak (0.20 ≤ *d* < 0.50), medium (0.50 ≤ *d* < 0.80), and large (0.80 ≤ *d*), while *r* strength of effect size was interpreted as weak (0.10 ≤ *r* < 0.30), medium (0.30 ≤ *r* < 0.50), and large (0.50 ≤ *r*) (Cohen 1992).

## Results

### Reproducibility of the acute HCF impact at rest

There were no significant differences in baseline parameters between the LCF and HCF trials (Table 1). Table 2 presents the results of the GLMM with random effects for baseline levels of all resting variables, measured at 1 h after LCF and HCF consumption. Cardiovascular and blood parameters, as well as psychological states in response to LCF and HCF at rest, did not differ between the two trials.

**Table 1:** Baseline states

	LCF	HCF	*P*-value	Effect size
*Cardiovascular parameters*				
Heart rate, bpm	61 [58–68]	63 [58–68]	0.73	*r* = 0.08
Blood pressure				
Systolic, mmHg	113 ± 10	118 ± 10	0.18	*d* = 0.46
Diastolic, mmHg	74 ± 5	75 ± 6	0.61	*d* = 0.14
Mean, mmHg	87 ± 5	89 ± 7	0.29	*d* = 0.33
*Blood parameters*				
Glucose, mg/dL	85 ± 7	83 ± 7	0.36	*d* = 0.22
Lactate, mM	1.5 [1.4–1.8]	1.6 [1.3–1.8]	0.37	*r* = 0.21
Hemoglobin, g/dL	15.7 ± 1.3	15.5 ± 1.3	0.43	*d* = 0.14
Hematocrit, %	48.7 ± 2.7	48.1 ± 2.9	0.42	*d* = 0.21
TBARS, μM	7.77 ± 3.52	6.78 ± 3.38	0.26	*d* = 0.29
BDNF, pg/mL	336 [209–547]	228 [178–564]	0.45	*r* = 0.18
*Psychological states*				
Visual analog scales, 0–100mm				
Mental fatigue	30 [7–45]	19 [5–54]	0.12	*r* = 0.36
Ability to concentrate	66 ± 20	65 ± 22	0.76	*d* = 0.05
Motivation	65 ± 20	71 ± 20	0.15	*d* = 0.31
Comfort	63 ± 23	73 ± 21	0.19	*d* = 0.45
Irritation	6 [0–15]	7 [1–16]	0.80	*r* = 0.06
Felt arousal scale, 1–6	3 [2–3]	3 [2–3]	0.25	*r* = 0.27
*Cognitive function, color-word Stroop task*				
Congruent task				
Averaged reaction time, ms	669 ± 84	669 ± 76	0.96	*d* = 0.01
SD of reaction time, ms	101 ± 39	95 ± 26	0.43	*d* = 0.20
Error, *n*	0 [0–1]	0 [0–0]	0.93	*r* = 0.02
Neutral task				
Averaged reaction time, ms	692 ± 88	678 ± 91	0.32	*d* = 0.16
SD of reaction time, ms	83 [70–103]	86 [80–98]	0.47	*r* = 0.17
Error, *n*	0 [0–0]	0 [0–1]	0.08	*r* = 0.41
Incongruent task				
Averaged reaction time, ms	748 ± 104	755 ± 120	0.65	*d* = 0.06
SD of reaction time, ms	104 [76–159]	108 [82–139]	0.98	*r* = 0.01
Error, *n*	0 [0–1]	0 [0–1]	0.48	*r* = 0.17
Reverse-Stroop interference score, %	6.2 [4.0–10.6]	10.5 [4.9–13.0]	0.06	*r* = 0.44

Values are mean ± standard deviation (SD) or median [interquartile range (IQR)]. *LCF*, low-cocoa flavanol trial; *HCF*, high-cocoa flavanol trial; *TBARS*, Thiobarbituric acid reactive substances; *BDNF*, Brain-derived neurotrophic factor

**Table 2:** Resting parameters 1-h after low- (LCF) and high-cocoa flavanol (HCF) consumption

		LCF	HCF	*P*-value	Effect size
*Cardiovascular parameters*					
Heart rate, bpm		55 [51–60]	57 [53–64]	0.31	*r* = 0.36
Blood pressure					
Systolic, mm Hg		119 ± 10	122 ± 10	0.33	*d* = 0.30
Diastolic, mm Hg		76 ± 8	78 ± 8	0.39	*d* = 0.20
Mean, mm Hg		90 ± 8	92 ± 8	0.32	*d* = 0.26
*Blood parameters*					
Glucose, mg/dL		81 [79–86]	82 [78–86]	0.62	*r* = 0.17
Lactate, mM		1.6 ± 0.3	1.6 ± 0.3	0.88	*d* = 0.07
Hemoglobin, g/dL		15.6 ± 1.1	15.4 ± 1.5	0.41	*d* = 0.16
Hematocrit, %		49 ± 3	50 ± 3	0.82	*d* = 0.06
Relative change in plasma volume, %		103 [99–104]	103 [101–104]	0.34	*r* = 0.34
Plasma TBARS, μM		5.7 [4.4–9.6]	7.1 [3.7–10.0]	0.27	*r* = 0.21
Plasma BDNF, pg/mL		594 [399–841]	435 [331–928]	0.69	*r* = 0.05
*Psychological states*					
Visual analog scales, 0–100mm					
Mental fatigue		25 [12–55]	18 [7–44]	0.64	*r* = 0.19
Ability to concentrate		66 ± 20	68 ± 22	0.62	*d* = 0.11
Motivation		64 ± 24	66 ± 22	0.89	*d* = 0.11
Comfort		25 [16–42]	22 [11–37]	0.33	*r* = 0.11
Irritation		4 [0–14]	6 [0–20]	0.17	*r* = 0.11
Felt arousal scale, 1–6		3 [3–4]	3 [3–3]	0.62	*r* = 0.24
*Cognitive function, color-word Stroop task*					
Congruent task					
Averaged reaction time, msec		675 ± 78	664 ± 78	0.36	*d* = 0.15
SD of reaction time, msec		97 ± 27	94 ± 39	0.78	*d* = 0.10
Error, *n/20*		0 [0–0]	0 [0–0]	0.42	*r* = 0.24
Neutral task					
Averaged reaction time, msec		698 ± 83	687 ± 74	0.37	*d* = 0.13
SD of reaction time, msec		91 ± 29	81 ± 24	0.27	*d* = 0.38
Error, *n/20*		0 [0–1]	0 [0–0]	0.07	*r* = 0.39
Incongruent task					
Averaged reaction time, msec		774 ± 146	731 ± 101	**0.02**	***d*** **= 0.34**
SD of reaction time, msec		98 [71–136]	106 [82–120]	0.83	*r* = 0.08
Error, *n/20*		1 [0–1]	1 [0–1]	0.82	*r* = 0.09
Reverse-Stroop interference score, %		6.2 [3.2–15.5]	4.6 [1.2–11.4]	**< 0.01**	***r*** **= 0.50**

Values are mean ± standard deviation (SD) or median [interquartile range (IQR)]. A generalized linear mixed model (GLMM) with random effects for baseline levels (see Table 1) was used to analyze. *TBARS*, Thiobarbituric acid reactive substances; *BDNF*, Brain-derived neurotrophic factor

Regarding cognition, HCF consumption was associated with a shorter average reaction time on the incongruent task at rest than LCF consumption (*P* = 0.02, *d* = 0.34). Likewise, HCF consumption resulted in a greater reduction in the reverse-Stroop interference score at rest (*i.e.*, better inhibitory executive processing) than LCF consumption (*P* < 0.01, *r* = 0.50).

### Impact of HCF on cognitive fatigue during exercise

HR (LCF 156 [147–166] *vs.* HCF 157 [150–166] bpm, *P* = 0.47, *r* = 0.18) and RPE (LCF 16 (14–16) *vs.* HCF 15 (13–17), *P* = 0.49, *r* = 0.16) during prolonged cognitive load combined with aerobic exercise were similar between the LCF and HCF trials. Blood parameters measured immediately after the combined prolonged cognitive load and aerobic exercise did not differ significantly between the two trials (Table 3). Additionally, there were no significant differences in psychological states, as measured by the VAS and FAS, immediately after the prolonged cognitive load combined with aerobic exercise between the two trials (Table 4).

**Table 3:** Blood parameters following aerobic exercise with prolonged cognitive load protocol

	LCF	HCF	*P*-value	Effect size
Glucose, mg/dL	81 [76–93]	84 [75–88]	0.71	*r* = 0.08
Lactate, mM	3.1 [2.5–3.5]	2.9 [2.2–4.0]	0.85	*r* = 0.13
Hemoglobin, g/dL	16.7 ± 1.1	16.7 ± 1.2	0.91	*d* = 0.02
Hematocrit, %	50 ± 3	50 ± 3	0.46	*d* = 0.16
Relative change in plasma volume, %	96.2 [93.6–99.0]	96.2 [94.1–99.5]	0.60	*r* = 0.29
Plasma TBARS, μM	6.4 ± 2.6	6.7 ± 3.1	0.60	*d* = 0.11
Plasma BDNF, pg/mL	839 [575–993]	562 [284–801]	0.16	*r* = 0.40

Values are mean ± SD or median [IQR]. A generalized linear mixed model (GLMM) with random effects (see Table 1) for baseline levels was used to analyze

**Table 4:** Psychological states following aerobic exercise with prolonged cognitive load protocol

	LCF	HCF	*P*-value	Effect size
Visual analog scales, 0–100mm				
Mental fatigue	64 [42–75]	62 [38–79]	0.98	*r* = 0.16
Ability to concentrate	64 ± 21	61 ± 23	0.70	*d* = 0.13
Motivation	60 ± 26	59 ± 25	0.74	*d* = 0.06
Comfort	44 ± 21	45 ± 19	0.94	*d* = 0.03
Irritation	10 [0–43]	20 [4–57]	0.37	*r* = 0.18
Felt arousal scale, 1–6	4 [4–]	4 [3–5]	0.31	*r* = 0.36

Values are mean ± SD or median [IQR]. A GLMM with random effects for baseline levels (see Table 1) was used to analyze

**Fig. 2:**
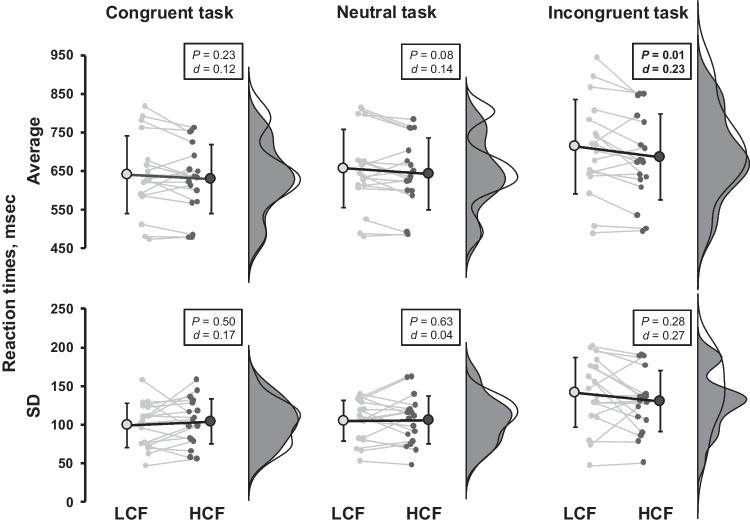
Reaction time during the 50-min color-word Stroop task (CWST) under prolonged cognitive load combined with aerobic exercise in low cocoa flavanol (LCF) (white/gray) and high cocoa flavanol (HCF) (black) trials. Raincloud plots show the distribution of reaction time data. Small circles represent individual data points, and large circles indicate the mean ± standard deviation (SD). The top panel shows the average reaction time over the 50-min collection period, while the bottom panel shows the SD during the same period

**Fig. 3:**
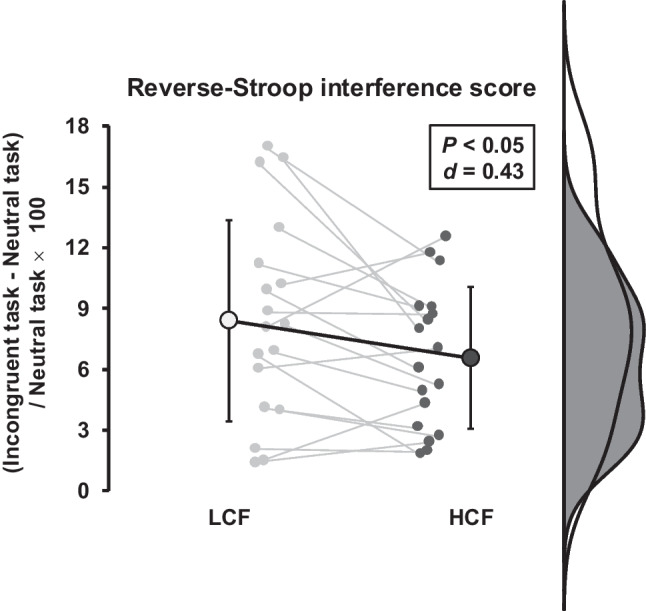
Reverse-Stroop interference score during prolonged cognitive load combined with aerobic exercise in LCF (white/gray) and HCF (black) trials. Raincloud plots show the distribution of reverse-Stroop interference scores. Small circles represent individual data points, and large circles indicate the means ± SD

In the 50-min CWST, HCF consumption did not affect response accuracy (Table 5). However, the average reaction time on the incongruent task throughout the 50-min cognitive load in conjunction with aerobic exercise was shorter in the HCF trial compared to the LCF trial (*P* = 0.01, *d* = 0.23; Fig. 2). Similarly, the reverse-Stroop interference score throughout the 50-min cognitive load combined with aerobic exercise was lower in the HCF trial than in the LCF trial (*P* < 0.05, *d* = 0.43; Fig. 3).

**Table 5:** The number of errors for the 50-min color-word Stroop task throughout aerobic exercise with prolonged cognitive load protocol

The number of errors	LCF	HCF	*P*-value	Effect size
Congruent task, *n/200*	1 [0–3]	1 [0–2]	0.60	*r* = 0.18
Neutral task, *n/200*	1 [1–2]	1 [0–3]	0.54	*r* = 0.08
Incongruent task, *n/200*	5 [4–9]	5 [2–15]	0.64	*r* = 0.09

Values are median [IQR]. A GLMM with random effects for baseline levels (*i.e.*, 5-min color-word Stroop task, see Table 1) was used to analyze

## Discussion

In the present study, we investigated the impact of HCF on decision-making quality and inhibitory executive processes during prolonged cognitive load in conjunction with aerobic exercise. Additionally, we measured psychological and physiological parameters to explore the underlying mechanism(s). Contrary to our hypothesis, HCF consumption failed to impact the increased RPE and state mental fatigue in response to prolonged cognitive load during aerobic exercise, thereby resulting in no changes in other psychological states. Nevertheless, in line with our hypothesis, HCF consumption resulted in shorter reaction times in the incongruent task and improved reverse-Stroop interference score during prolonged cognitive load in conjunction with aerobic exercise. These findings suggest that HCF may be capable of improving the speed of decision-making and inhibitory executive processes during prolonged cognitive activity and aerobic exercise. Therefore, HCF supplementation before sports matches can be a new feasible nutritional strategy for improving decision-making speed, potentially ensuring superior sporting performance. However, the precise mechanism through which HCF improves behavioral performance and inhibitory executive processes remains elusive.

HCF has been shown to reduce mental fatigue induced by prolonged cognitive load (Scholey et al. 2010). Conversely, HCF did not mitigate mental fatigue enhanced by aerobic exercise (Tsukamoto et al. 2018). These findings suggest that the efficacy of HCF varies between prolonged cognitive load and that enhanced by aerobic exercise. In a previous study, we found that prolonged cognitive load in conjunction with aerobic exercise can enhance RPE and mental fatigue compared to aerobic exercise alone (Tsukamoto et al. 2022), suggesting that prolonged cognitive load adds to the mental fatigue induced by aerobic exercise. Actually, mental fatigue was increased after prolonged cognitive load and aerobic exercise in this study, as compared with baseline states (see Tables 1 and 4, additional analysis using a generalized linear model [time*trial]; main effect for time *P* < 0.01). However, in this study, HCF did not reduce RPE or mental fatigue in response to the combined prolonged cognitive load and aerobic exercise. Thus, HCF did not substantially reduce mental fatigue enhanced by prolonged cognitive load during aerobic exercise. It is possible that the HCF-mediated reduction in mental fatigue during prolonged cognitive activity (Scholey et al. 2010) was obscured by the strong negative impact of exercise on psychological states such as RPE and mental fatigue.

We previously demonstrated that CWST reaction time and the reverse-Stroop interference score were reduced at rest 1 h after HCF consumption despite no detectable changes in resting psychological states (Tsukamoto et al. 2018). Similarly, this study showed that HCF consumption shortened reaction time in the incongruent task and improved reverse-Stroop interference score 1 h post-consumption, just before the onset of prolonged cognitive load during aerobic exercise. Furthermore, throughout the 50-min cognitive load combined with aerobic exercise, the average reaction time in the incongruent task and the reverse-Stroop interference score were better in the HCF trial than in the LCF control trial. These findings suggest that consuming HCF 1 h before training and/or sports matches may improve decision-making during aerobic exercise, potentially enhancing athletic performance. Notably, HCF has also been shown to enhance physical fitness in professional soccer players (González-Garrido et al. 2017), further supporting its role as a beneficial sports supplement when consumed before exercise or competition.

We explored the potential physiological mechanism(s) underlying the findings of this study; however, the exact mechanism remains unknown. Exaggerated oxidative stress may contribute to acute cognitive decline (Ando et al. 2020). Compared to aerobic exercise alone, psychological stress—such as performing a color-word Stroop task during moderate-intensity aerobic exercise— can increase oxidative stress (Huang et al. 2010). HCF is known as an antioxidant nutrient (Keen et al. 2005) and contains (+)-catechin, which has been shown to reduce oxidative stress in human plasma samples by decreasing TBARS levels enhanced by 2,2’-azobis-(2-amidinopropane) clorhidrate or 2,2’-azobis(2,4-valeronitrile) (Lotito and Fraga 1998). However, in this study, plasma TBARS levels—a marker of oxidative stress—were not affected 1 h after HCF consumption compared to LCF consumption. Similarly, HCF consumption did not alter TBARS levels immediately after the prolonged cognitive load in conjunction with aerobic exercise. Furthermore, HCF did not influence changes in BDNF levels either 1 h after consumption or immediately after the prolonged cognitive load combined with aerobic exercise. BDNF is partially secreted by brain endothelial cells (Wang et al. 2006). Schroeter et al. (2006) reported that brachial endothelial function improves 1–2 h after ingestion of epicatechin, a major component of HCF; however, whether this effect extends to cerebral endothelial function remains unclear. Regarding cerebral blood flow regulation, Francis et al. (2006) demonstrated that HCF can acutely enhance neurovascular coupling across gray matter and maintain this effect for up to 4 h, suggesting that the benefits of HCF on cerebral blood flow regulation may persist for a few hours. During aerobic exercise alone, the motor cortex is activated while the dorsolateral prefrontal cortex is deactivated to prioritize blood flow to motor regions (Fontes et al. 2020). In contrast, the dorsolateral prefrontal cortex is activated during the CWST (Wagner et al. 2001). Since both the motor and dorsolateral prefrontal cortices are likely activated during concurrent cognitive tasks and aerobic exercises, HCF-enhanced neurovascular coupling may play a key role in the observed effects.

We previously demonstrated that a single intake of HCF can improve the inhibitory executive process across a 160-min experimental protocol, which included 30 min of aerobic exercise (Tsukamoto et al. 2018). Based on this study, HCF may sustain cognitive improvements during/after aerobic exercise for a few hours; however, the bioavailability and optimal timing/frequency of HCF intake for improving inhibitory executive process remains unknown. Incidentally, approximately 500 mg dose of HCF appears more effective for reducing mental fatigue and cognition than either 900 mg or LCF (Scholey et al. 2010). Increasing the single dose of cocoa flavanols beyond 500 mg does not appear to be a viable strategy for prolonging the cognitive benefits of HCF in response to prolonged cognitive load combined with aerobic exercise.

We assumed that mental fatigue accumulating during sports matches impairs the speed and accuracy of decision-making, thereby reducing performance (Smith et al. 2016; Habay et al. 2021). In football, goal-scoring rates tend to increase in the later stages of a match, when fatigue is also more pronounced (Alberti et al. 2013), potentially due to compromised cognitive perception (Reilly 1997). In this study, HCF did not reduce subjective mental fatigue but did shorten the average reaction time during the incongruent task by −27 ± 47 ms (*d* = 0.23). Although the effect size of HCF on reaction time during 50 min of prolonged cognitive load in conjunction with aerobic exercise was weak, even a modest improvement can be meaningful in sports performance. For instance, Japan defeated Spain 2–1 in the 2022 FIFA World Cup in Qatar. In that match, a critical goal was awarded after the video assistant referee confirmed the ball remained in play by just 1.88 mm—illustrating how even minimal improvements in decision-making speed can be crucial in high-level competition.

This study has some limitations. First, we previously reported that prolonged cognitive activity impairs the speed of decision-making and the inhibitory executive process during aerobic exercise in males but not in females (Tsukamoto et al. 2022). Therefore, only male participants were recruited for this study. It is known that cognitive function, including CWST score, can vary across menstrual cycle phases (Hatta and Nagaya 2009). Specifically, differences in baseline cognitive characteristics during the follicular and luteal phases may influence acute cognitive responses to cognitive fatigue or aerobic exercise (Ishihara et al. 2021). Conversely, Grissom and Reyes (2019) argued that evaluating sex differences in executive function may not be essential for generalizability, given the minimal sex differences in decision-making. However, physiological brain-related changes may still differ between sexes (Marley et al. 2020). Further research should examine the effects of HCF on decision-making quality and inhibitory executive processes in female participants, considering potential influences of endogenous estrogen fluctuations across the menstrual cycle. Second, this study measured only plasma TBARS levels as a biomarker of oxidative stress (Lotito and Fraga 1998) based on prior findings that lower circulating TBARS is associated with better CWST scores (Ji et al. 2023). However, brain-specific oxidative stress levels remain unknown. Additionally, oxidative stress can be directly/indirectly assessed using alternative biomarkers such as ascorbate free radicals (Stacey et al. 2025) and 8-isoprostane (Huang et al. 2010), which were not measured in this study. Similarly, brain BDNF content was not assessed. Finally, possibly due to limited statistical power, the neutral task scores did not differ significantly between the two trials. We conducted an a priori sample size calculation based on the changes in the inhibitory executive process following a prolonged CWST during aerobic exercise (Tsukamoto et al. 2022). However, the expected effect size may have been small to detect HCF-related differences. Nonetheless, this statistical limitation is unlikely to alter the overall conclusions of this study.

## Conclusion

HCF consumption unconsciously (*i.e.*, no impact on psychological states) improved the speed of decision-making and inhibitory executive process during prolonged cognitive load in conjunction with aerobic exercise. In situations where sports demand frequent and sustained cognitive engagement, HCF may serve as an effective nutritional strategy to enhance cognitive function during aerobic exercise.

## Data Availability

The data supporting the findings of this study are available from the corresponding author, H.T., upon reasonable request.

## Abbreviations

CWST: Color-word Stroop task
HCF: High cocoa flavanol
LCF: Low cocoa flavanol
BDNF: Brain-derived neurotrophic factor
TBARS: Thiobarbituric acid reactive substances
VO_2_ peak: Peak oxygen uptake
HR: Heart rate
VAS: Visual analog scale
FAS: Felt arousal scale
RPE: Rating of perceived exertion
